# High-quality genomes of *Paenibacillus* spp. RC334 and RC343, isolated from a long-term forest soil warming experiment

**DOI:** 10.1128/MRA.00371-23

**Published:** 2023-08-28

**Authors:** Claire E. Kitzmiller, Wyatt C. Tran, Brendan Sullivan, Florencia Cortez, Mallory Choudoir, Rachel Simoes, Nipuni Dayarathne, Kristen M. DeAngelis

**Affiliations:** 1 Department of Microbiology, University of Massachusetts, Amherst, Massachusetts, USA; 2 Department of Plant and Microbial Biology, North Carolina State University, Raleigh, North Carolina, USA; The University of Arizona, Tucson, Arizona, USA

**Keywords:** soil microbiology, climate change

## Abstract

*Paenibacillus* spp. RC334 and RC343 were isolated from heated soil in a long-term soil warming experiment. Both genomes were 5.98 Mb and assembled as a single contig. We describe the assembly and annotation of the two high-quality draft genomes for these isolates here.

## ANNOUNCEMENT

Soil microbes have the potential to mitigate the impact of global warming, but we don’t understand how they respond to long-term warming (1, 2). We sequenced bacterial genomes isolated from the Harvard Forest soil warming experiment (2) to understand the genetic profile of these novel species.

*Paenibacillus* spp. RC334 and RC343 were isolated in 2022 on Actinobacteria Isolation Agar (3) with 100 mg L-1 cycloheximide from mineral soil from a heated soil plot (43.54N 72.18 W), collected 10 cm below the surface, at an elevation of 355 m, using a steel corer. The isolates were streaked, and single colonies were grown in 10% tryptone soy broth media at 30°C shaking at 150 rpm until OD of 0.5. gDNA was extracted by CTAB (4) for RC343 and using the Blood & Tissue DNEasy Kit (Qiagen) following the manufacturer’s instructions for RC334. Libraries were prepared with the Ligation Sequencing Kit SQK-LSK-109, and samples were multiplexed using the Native Barcoding Expansion Kit EXP-NBD104. Oxford Nanopore sequencing was performed at SeqCenter (Pittsburgh, PA) using R9 flow cells (R9.4.1). High accuracy base calling with Guppy v4.5.4 was used to achieve Q20 performance.

The genomes were assembled, annotated, and analyzed as part of the Bioinformatics Lab (MICROBIO 590B) course at the University of Massachusetts Amherst (5). FiltLong (6) was run to remove low-quality reads and specified a 40× coverage for RC334 and for RC343. The genomes were assembled *de novo* using Flye (7), and then Minimap2 (8) mapped the genome and completed pairwise alignment. Racon (9) created a genomic consensus, and Medaka (10) polished consensus sequences. Quast (11) and CheckM (12) were then used to assess the quality of the assembly. The genome assemblies for RC334 and RC343 are both of high quality (Table 1) (13).

**TABLE 1:** Genome assembly details from Quast

Features	RC334	RC343
Total base pairs in the assembly (bp)	183,313,012	244,003,914
Assembled genome size (bp)	5,979,552	5,982,416
Fold-coverage (total bp/genome size)	30.6	40.8
Assembly N50 (bp)	5,979,552	5,982,416
Assembly N75 (bp)	5,979,552	5,982,416
Number of contigs	1	1
G + C content (%)	46.72	46.71
Completion (%)	99.12	96.8
Contamination (%)	0.07	0.07

The final assemblies were uploaded to KBase for analysis and annotation (14). All apps were run on the default settings unless otherwise indicated. Genomes were annotated using Prokka (Annotate Assembly and Re-annotate Genomes with Prokka—v1.14.5) (15) and classified using GTDB-Tk v1.7.0 (16) which assigns a taxonomic classification to the organism using domain-specific, concatenated proteins. Both genomes’ domain is Bacteria, the phylum is Firmicutes, the class is Bacilli, the order is Paenibacillales, the family is Paenibacillaiceae, the genus is *Paenibacillus,* and the species is *Paenibacillus terrae_A*.

**Fig 1:**
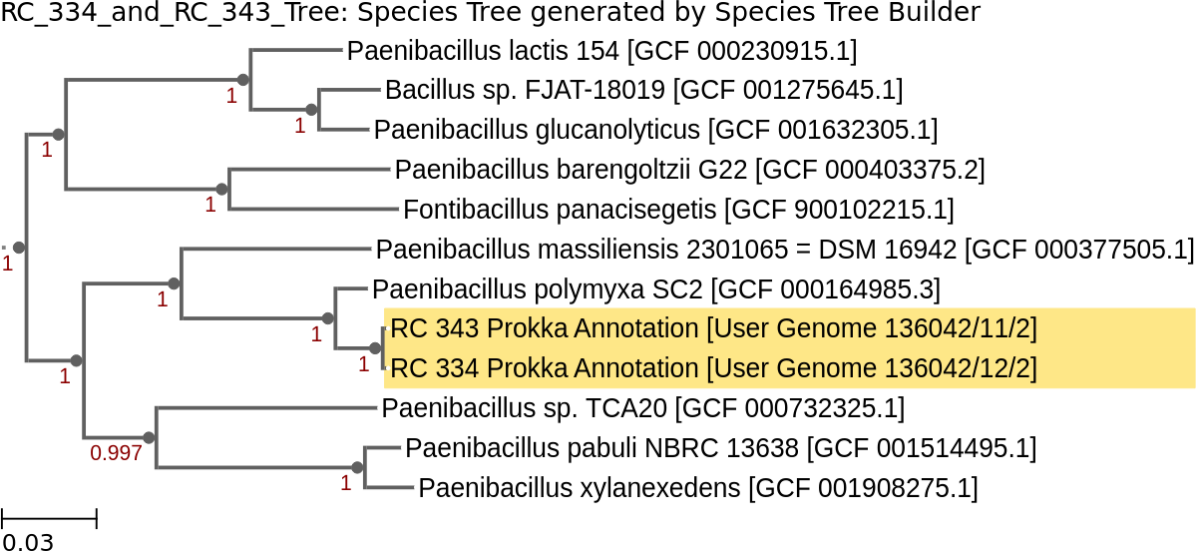
Phylogenetic tree based on 49 core genes. The phylogenetic tree was created on KBase (Insert Genome into SpeciesTree) (19) using a set of 49 core, universal genes defined by COG.

The nearest neighbor for both genomes was identified to be *Paenibacillus polymyxa* SC2 using the phylogenetic tree made with a comparison of 49 clusters of Orthologous Groups (COG genes) (Fig. 1). Compute Average Nucleotide Identity (ANI) with FastANI v0.1.3 (17, 18) calculated the Average Nucleotide Identity between *P. polymyxa* SC2 and RC334 and RC343 to be 85.4% and 85.3%, respectively. RC334 and RC343 both had 94.2% ANI with a user *P. terrae* (GCF_000235585.1_assembly) genome assembly and annotation. When RC343 was compared to RC334, the ANI estimate was 99.95%. Since the ANI number is less than 95%, it is likely that these compared genomes are from different species than *P. polymyxa* and *P. terrae*. Further research into these isolates may provide deeper insight into the role of *Paenibacillus* sp. in microbial climate change responses.

## Data Availability

The 16S rRNA gene sequence accession number for RC343 is OQ547097. The 16S rRNA gene sequence accession number for RC334 is OQ547098. The raw whole-genome sequence reads are available in GenBank under the BioProject accession number PRJNA949990. The BioSample accession number for RC334 is SAMN33990111 and for RC343 is SAMN33990112. The Sequence Read Archive (SRA) accession number for RC334 is SRR24019814 and for RC343 is SRR24019813. The draft genome reference number for RC334 is NZ_CP125370.1 and for RC343 is CP125371.1.
